# Detection and impact estimation of social bots in the Chilean Twitter network

**DOI:** 10.1038/s41598-024-57227-3

**Published:** 2024-03-19

**Authors:** Marcelo Mendoza, Eliana Providel, Marcelo Santos, Sebastián Valenzuela

**Affiliations:** 1 Department of Computer Science, Pontificia Universidad Católica de Chile, Santiago, Chile; 2 School of Informatics Engineering, Universidad de Valparaíso, Valparaíso, Chile; 3 School of Communications, Universidad Diego Portales, Santiago, Chile; 4 School of Communications, Pontificia Universidad Católica de Chile, Santiago, Chile

**Keywords:** Social bots, Bot detection, Political campaigns, Computer science, Information technology

## Abstract

The rise of bots that mimic human behavior represents one of the most pressing threats to healthy information environments on social media. Many bots are designed to increase the visibility of low-quality content, spread misinformation, and artificially boost the reach of brands and politicians. These bots can also disrupt civic action coordination, such as by flooding a hashtag with spam and undermining political mobilization. Social media platforms have recognized these malicious bots’ risks and implemented strict policies and protocols to block automated accounts. However, effective bot detection methods for Spanish are still in their early stages. Many studies and tools used for Spanish are based on English-language models and lack performance evaluations in Spanish. In response to this need, we have developed a method for detecting bots in Spanish called Botcheck. Botcheck was trained on a collection of Spanish-language accounts annotated in Twibot-20, a large-scale dataset featuring thousands of accounts annotated by humans in various languages. We evaluated Botcheck’s performance on a large set of labeled accounts and found that it outperforms other competitive methods, including deep learning-based methods. As a case study, we used Botcheck to analyze the 2021 Chilean Presidential elections and discovered evidence of bot account intervention during the electoral term. In addition, we conducted an external validation of the accounts detected by Botcheck in the case study and found our method to be highly effective. We have also observed differences in behavior among the bots that are following the social media accounts of official presidential candidates.

## Introduction

In today’s information age, billions of people all over the world use social media platforms to access content. Despite the increased access to information, people’s understanding of important issues like politics and economics has not improved at the same rate^1^. One crucial factor contributing to this paradox is the challenge faced by users in distinguishing reliable, high-quality information sources from unreliable, low-quality ones^2^. Studies show that social platforms enable the dissemination of rumors, conspiracy theories, and other forms of misinformation, which can spread rapidly^3^. Social media have also created a diverse and fragmented media landscape, which has resulted in the emergence of “competing, often chaotic, voices”^4^. Moreover, social platforms have been exploited to disseminate political propaganda and other forms of disinformation^5^.

Automating activity on social networks is an effective way to increase the visibility of misinformation and propaganda^6^. This includes behaviors such as spamming and trolling, which promote specific ideas for the benefit of specific agendas. Spamming is done by inflating the interaction cues of particular users or by repeating slogans and hashtags to artificially increase the visibility of propaganda content. On the other hand, trolling is used to discredit certain individuals and their ideas, leading to incivility and polarization^7^. Trolling is often expressed through hate speech and can undermine specific groups in society. Automated spamming is also used to overpopulate hashtags with contentious action, where Twitter users gather to coordinate social mobilization, adding so much noise to the data that it becomes useless^8^.

To respond to the problem of online misinformation, spamming, trolling, and hate speech, strict usage protocols have been defined. Bot-blocking policies have also been implemented to eliminate highly automated accounts. However, due to these policies, a new type of automated account known as social bots has emerged. These bots attempt to blend in with humans, making them difficult to detect. Social bots alternate between periods of inactivity and periods of automated activity during campaigns^9^. By mimicking human behavior, they attract more followers and effectively blend in with humans^10^. During campaigns, they send out thousands of messages and work in coordination with other bots^11^.

The weaponization of automated activity on social networks has become a social problem. Therefore, it is crucial to create models that can identify bot activity. However, detecting bots is a complex task^12^. To address this issue, the scientific community has proposed algorithmic approaches based on machine learning for detecting bots^13^.

Some of these methods have advanced to a higher level of maturity^14^. Nonetheless, bot detection in low-resource languages has not been as successful as in English-speaking countries^15^. For example, in Spanish-speaking countries, a significant portion of bot impact analysis still relies on classifiers trained with data that have not been systematically validated in foreign languages^15^. According to^16^, one of the main issues with bot detectors is their high false positive rate. To overcome this challenge, we aim to develop a bot detector with specific language processing capabilities in Spanish.

We introduce Botcheck, a bot detector specifically designed for detecting bots in the Spanish language. We have selected various features commonly used in addressing this issue, adapting to Spanish those features that are language-dependent. Botcheck also defines novel characteristics based on account usage patterns and from the user profile. In total, Botcheck considers 265 descriptors per account. To improve the accuracy of our bot detector, we explored several machine-learning methods based on annotated data in Spanish. We selected the best-performing model, which outperforms other competitive methods.

As a case study, we applied Botcheck to the Twitter network in Chile during the 2021 presidential election campaign. We conducted an external validation of the accounts detected by Botcheck and found our method to be highly effective. We observed that Botcheck was able to detect automated activity during the campaign. We have also observed differences in behavior among the bots that are following the social media accounts of official presidential candidates.

### Contributions

The contributions of this work are:

- We present Botcheck, a machine learning-based method designed to identify bots on Spanish Twitter accounts.
- Our research involves an analysis of the results of Botcheck, which indicates which characteristics are most valuable for distinguishing between bots and humans. The analysis provides insights into the key features that reveal automated behavior, which are used to explain the outputs of the model.
- To evaluate the effectiveness of Botcheck, we analyzed the 2021 Chilean presidential campaign period. Our analysis revealed the presence of bots, many of which supported specific candidates and trolled others. Furthermore, our findings highlight disparities in bot usage between politicians.

### Roadmap

The rest of the work is organized as follows. In “Related work”, we review related work. In “Botcheck: features, data, and models”, we introduce Botcheck, defining the characteristics used by the classifier and the data on which it was trained. “Experimental results” presents the validation of Botcheck, comparing its performance with other methods. “Case study: 2021 Chilean presidential elections” presents the case study. Finally, in “Conclusion” we provide concluding remarks and outline future work.

## Related work

### Bot detection

In the last few years, the detection of bot accounts has been a topic of interest in social media research. This section reviews some of the most relevant and recent works on the subject.

Bot detection started many years ago with the intent to detect web spammers^6^. released social honeypots – i.e., accounts capable of attracting and unmasking spammers. The basic idea behind honeypots is that they are very simple bots, which are of no interest to ordinary users. As such, honeypot followers are expected to be other bots and spammers. By making a characterization of honeypot followers, the authors trained classifiers that could accurately detect spammers and other malicious accounts. Still using honeypots, the same authors later carried out a longitudinal study of 7 months of observation of spammers and content polluters on Twitter^17^. In the study, the authors measured the characteristics of spammers highlighting differences with legitimate users. Among the most relevant features, the study shows that spammers follow more accounts and also have bursts of new followees in short time frames. Also, malicious accounts tend to be young and to be active on the platform only for a limited time, being replaced by other accounts that perform a similar function during a campaign.

Botometer^18^ evaluates whether a human or a machine controls an account of Twitter. It corresponds to a machine learning-based system, which considers more than 1,000 features grouped into: Network features, User features, Friends features, Temporal features, Content features, and Sentiment features. Although it was designed to work with English posts, it is also used with data in other languages. Botometer’s classifier uses Random Forest, an ensemble supervised learning method. Extracted features are leveraged to train seven different classifiers: one for each subclass of features and one for the overall score. To classify an account as either social bot or human, the model is trained with instances of both classes.

Botometer is used in various research works, such as the proposal of^19^ that researches the influence of social bots in Mexican Twitter posts associated with different trending topics. Their focus was specifically on the $\#$Tanhuato hashtag. They use two thousand tweets obtained from Twitter API. The purpose of the research was to determine the intention of the bot accounts and their impact on the diffusion of information. Their analysis shows that bots impacted the proliferation of disinformation among the community.

^20^ used Botometer to evaluate the impact of bots and trolls over Twitter posts associated with vaccination in Russia. They used 1.7 million tweets collected between 2014 and 2017^21^. compared the 2016 USA presidential campaign with the 2017 elections in Germany. Again, Botometer was used to identify accounts and discover the properties of these accounts, focusing the study on structural and functional properties. How can bot accounts influence information propagation was the topic studied by^22^. By comparing bots and human accounts, they studied the strategies used to spread information. They used data associated with natural disasters labeled with Botometer. Recently^23^, developed *Pegabot*, a “bot catcher” inspired on Botometer parameters, but considering local specificity for Portuguese-based messages. *Pegabot* stands on top of four dimensions: profile data, networked behavior, sentiment analysis and temporal patterns. The authors used Pegabot to analyze the Brazilian presidential elections, determining the presence of bots during the electoral campaign.

In addition to Botometer, there are other bot detection initiatives based on machine learning approaches^12^. approach the problem of spambot detection based on the codification of accounts using the so-called “digital DNA”, which, for a given user timeline, codifies the ’tweet’, ’reply’, and ’retweet’ actions as the ’A’, ’C’, and ’T’ bases, respectively. Bot detection relies on the comparison of the longest common substring between groups of accounts and the application of an unsupervised learning approach.

Related work showed that bots tend to interact synchronously to amplify certain events, thus showing different patterns concerning those of humans^24^. For this reason, informative embeddings for bot detection tasks should account not only for the structure of connections (who follows whom) but also for the interactions between them (who interacts with whom)^25^ addressed the bot detection task from this approach. Their proposal is independent of the language and uses semi-supervised learning, given the difficulty of obtaining a fully-labeled network as input data for training. Instead, the proposed approach learns to represent social connections. The user’s interactions enhance this representation by generating a proximity graph according to the distance between user embeddings. Account labeling was performed by a label propagation algorithm, where a reduced set of labels serves as seeds for label propagation through other nodes of the graph.

Another proposal is Infoshield^26^, a method based on information theory that aims to find duplicate text in social media, constructing micro-clusters of similar text. The study focuses on two domains: (i) Human Trafficking Detection and (ii) Social Media Bot Detection. The method has advantages such as being language and domain-independent^27^ study the performance of a small set of features in bot detection, emphasizing user metadata and derived secondary characteristics such as length of name and description. The authors emphasize that using a reduced number of features favors the scalability of the method without affecting task performance. Their approach, based on Random Forest, does not use language-dependent features, which makes them take advantage of cross-lingual and multilingual scenarios, as shown by^28^ when using this method in Twibot-20.

^29^ compared the performances of various standard machine learning methods in two bot detection-related problems: account-level classification and tweet-level classification. The authors argue that account-based methods require collecting historical data, making it difficult for these methods to scale in large networks. A tweet-level classifier would require less effort in historical data collection while offering better features in terms of scalability. The authors encode the tweets using GloVE^30^ and add side information extracted from the user profile metadata to an LSTM-based classifier. The authors show that this problem is more complex than the account-level classification. Using their LSTM model, the authors outperform other models in this task.

^31^ employed various pre-trained models to encode text in a bot detection system, with RoBERTa^32^, an adaptive version of the BERT embedding whose goal is to improve the performance in longer sequences, yielding the best results. Their method merges RoBERTa’s encodings with user profile metadata to create a concise representation of each account. These representations are then utilized in a fully dense neural network to identify which accounts are likely bots.

^33^ studied the performance of one-class classifiers in bot detection. One-class classification is used to work with target classes as anomalies, allowing work with unbalanced data where there are many examples of a background class (human) and only a few annotated accounts in the target class (bot). In this scenario, the model learns the background class and distinguishes anomalous examples from this class, favoring the detection of new types of bots. The authors show that binary classifiers deteriorate their performance when facing new types of bots. However, this deterioration is less when using one-class classifiers^34^, also noted that binary classifiers trained on a single class of bots deteriorate their performance when facing new bots. To deal with these difficulties^14^, created Botometer V.4, training an ensemble of specialized classifiers (ESC). Each base classifier was trained with accounts annotated on different types of bots. For example, Botometer V.4 considers astroturfers (bots of political propaganda), fake followers (bots of amplification of followers), financial bots (financial supporting bots), spambots (bots of production of repetitive content), and self-declared bots, among others. In addition, Botometer V.4 provides two types of scores, Universal and English-dependant. The authors show that the ensemble outperforms binary classifiers trained in a single bot class, arguing that this advantage is because each base classifier learns specific characteristics for each type of bot. The authors indicate several challenges for this method, highlighting that the cross-domain performance is highly sensitive to the datasets used to train each base classifier. We also note this limitation when evaluating Botometer V.4 on Spanish-based accounts.

### Bot detection in Spanish

Although the detection of bots in Spanish is a topic that has been studied from different perspectives, the literature is not as extensive as in the case of English. For instance^35^, explored the behavior of bot accounts during the 2017 Chilean presidential elections, working with data associated with eight presidential candidates in the first round of presidential elections in 2017. The authors conducted both manual and automatic analyses of suspicious accounts. The aim was to identify potential indicators of inauthentic activity during presidential debates. To validate their findings, they manually annotated 2,472 bot accounts. For the automatic detection phase, they utilized 61 features from user accounts, which were grouped into user, friendship, network, and temporal features. Additionally, they analyzed content, language, and sentiment features from the text of tweets. To create a training dataset and compare various machine learning models, the authors used Botometer outputs to annotate the data.

^36^ studied the presence of bots, the behavior and activity of bots accounts, and the differences with human accounts. They inferred the political affinity of the accounts, the sentiment in the tweets, and how the bots relate to the general 2019 Spanish elections. They used 5.8 million tweets from approximately 780 thousand unique users. The accounts were classified as human or bot using Botometer. In data analysis, they extracted features like the score associated with the sentiment of every tweet and topic mentions. Their study shows how bots are used in presidential debates to amplify and propagate specific ideas, amplifying the visibility of specific posts.

^37^ proposed an explainable approach for bot detection based on rules. The authors combine features from user accounts, tweet content, and network metadata. Regarding tweet content, the proposal pays attention to sentiment-based features, highlighting their contribution to this task. The model is evaluated on a dataset containing accounts in English belonging to the “genuine” dataset^38^ and then combined with Spanish-based accounts annotated by the authors. The authors show that the method can work with both types of accounts, highlighting the rules exploited using Random Forest. The authors conclude that another rule-based technique named contrast-pattern classification could achieve similar performance to Random Forest but with fewer rules.

Bot detection has been addressed in the Author Profiling (AP) Task at PAN 2019^39^, where they focus on solving whether an author is a bot or a human and if it is a human, to determine its gender. The task presents a dataset in Spanish and another dataset in English, considering only the content of the tweets, ruling out the use of profile-based features. In the 2019 competition, there were 54 teams, where 41 of them considered both languages and 13 only considered Spanish. For the Spanish case, the best result was obtained by^40^ using an SVM classifier, with features represented as char/word n-grams, using Tf-Idf for text vectorization. In addition to this work, the competition received other competitive proposals. We highlight three very competitive works. First^41^, worked with classic methods for text classification, considering text preprocessing, feature extraction, and classification. They emphasize that preprocessing is the most critical stage. They consider different criteria for selecting the *k* most relevant terms, such as Document Frequency, Frequently co-occurring entropy, and Information Gain. Finally, they study how the text structure differs between humans and bots. Their best results were obtained using {3, 5} char n-grams and using Document Frequency for selecting the most relevant terms^42^ worked with a deep learning model that combines a 2D-CNN (2D Convolutional Neural Network) with a fully connected neural network. They represent the text using FastText for the Spanish language. Using this pre-trained model, they obtain better results than other techniques such as word2vec, word n-grams, and char n-grams^43^, explored three text encoding techniques: Semantic Encoding based on word2vec (W2V), a Syntax-Modeling Encoding based on deep learning (SYN), and character Encoding based on deep learning (CHAR). The best result was obtained using a combination of W2V and SYN encoding, a representation conveying both syntactic and semantic information from text data^44^ used the dataset of AP at PAN 2019 to investigate which stylometry-based features are favorable to resolving the problem. That is, they present an approach that includes feature extraction, using 17 stylometric-based features. The best result based on these features was obtained with Random Forest. Recently^45^, revisited bot detection in Spanish using Isolation Forest^46^, a technique to deal with bots as an anomaly detection task. The authors evaluated the method in the 2020 Chilean Referendum, showing the presence of bots during the electoral campaign.

**Literature gap.** As a result of our research, we have identified a gap in the literature on Spanish-based bot detection methods. On the one hand, we have found that some methods^35,36^ are based on model outputs that have been pre-trained using English data. Given that linguistic features are crucial for detecting bots, it is not surprising that these methods may be biased toward the language of the users with whom they were trained. Furthermore, cultural, linguistic, and stylistic differences between English and Spanish can also affect the transferability of these models. For example^37^, create a bilingual dataset for bot detection using texts in English and getting features in Spanish using machine translation without providing evidence about the loss of information between both versions of the data. Recent studies have shown the weaknesses of transfer learning based on automatic bilingual translation, a factor attributable to linguistic information loss, misalignment of meanings, and cross-lingual transfer effects^47^. On the other hand, the PAN 2019 competition^39^ has addressed the issue of bot detection on anonymized accounts. However, several bot detection studies^25,48,49^ have shown that characteristics extracted from user profiles and interaction patterns are also critical for detecting bots. In addition, there is a lack of models in Spanish that use human-annotated data in languages other than English. To address this limitation^45^, propose a detection strategy based on one-class learning. This model detects bots based on anomaly detection but requires the apriori definition of a proportion of bots to establish a threshold for detecting such anomalies. All of these factors highlight a gap between the advancements in bot detection methods in English and Spanish. To address this gap, we developed Botcheck, a method trained on Spanish-based accounts annotated by humans in Twibot-20^28^, a large-scale multilingual dataset developed for benchmarking in bot detection. We selected various features used in bot detection models, adapting those that are language-dependent to Spanish. Additionally, we introduced a set of novel profile-based features for this task. This study shows that Botcheck outperforms competitive methods, including those based on deep learning.

### Ethics approval

The study received an exemption from ethical review by the Ethics and Research Safety Unit of the Pontifical Catholic University of Chile on October 2, 2023.

## Botcheck: features, data, and models

### Overall architecture

**Figure 1:**
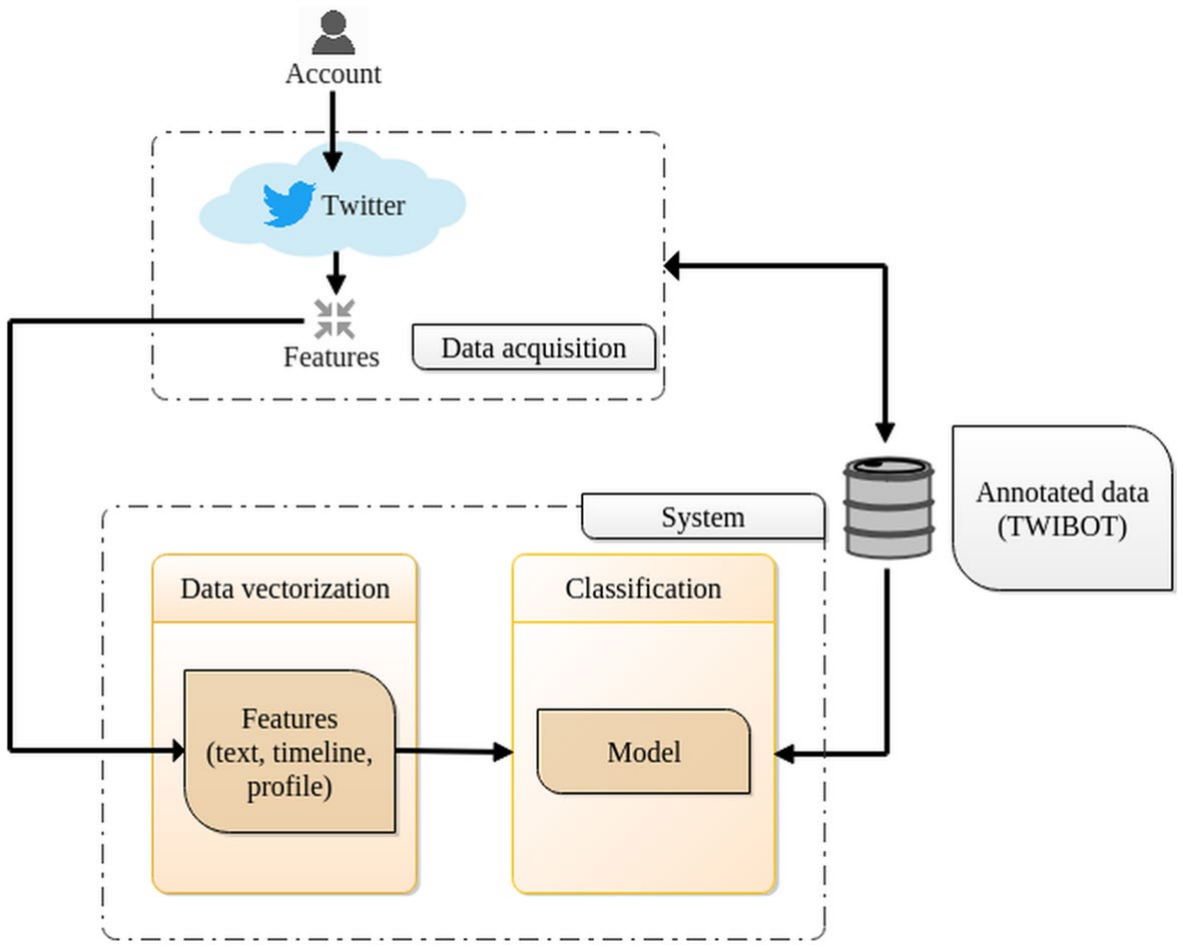
High-level architecture of our system, whose main components appear in the boxes. Our system requires two inputs: (i) Features obtained from Twitter, and (ii) Annotated data (accounts) for the classifier.

In this section, we provide an overview of our system for bot detection, whose general architecture is shown in Fig. 1, outlining our method and the rationale for our design choices.

As depicted in Fig. 1, our system comprises a data acquisition component that is responsible for gathering data from the Twitter API. This component collects tweets and meta-level features of the account using an account as input. It is utilized for both annotated accounts and unseen accounts. The system consists of two primary components: (i) the vectorization module that computes a feature vector for each account, and (ii) a model that predicts whether the account is a bot or not.

During model training, we used Twibot-20^28^, which is a dataset for detecting Twitter bots. Twibot-20 covers various types of bots and authentic users and supports binary classifications of individual users as well as community-aware approaches. This benchmark encompasses accounts in multiple languages, including 6,511 Spanish-written tweet accounts (The authors of Twibot declare that due to privacy issues they are not directly posting the dataset. Researchers interested in using the dataset should contact shangbin@cs.washington.edu to obtain permission to download the dataset for research purposes. For further details on the data please visit: https://github.com/BunsenFeng/TwiBot-20). The annotated data is divided into two categories: bot or human and includes user IDs, enabling us to retrieve user-profile features and tweet timelines using the Twitter API.

### Features

Feature engineering remains a prominent method for detecting bots due to its ability to provide interpretable models^9^. This process not only aids in understanding the specific behavioral patterns indicative of automated actions but also is vital in justifying the decisions made by the classifier. Such justification is especially important when reporting to platforms like Twitter, where it could lead to the suspension of an account.

We have selected features previously introduced in Botometer^14^. Among these, those that are language-dependent have been adapted to Spanish using linguistic resources and Spanish-based NLP tools. Furthermore, we have introduced novel features. All of these features take into account three sources of information, as shown in Table 1.

The first source allows us to analyze the content of the 100 most recent tweets of a given account. This source provides distributional features because each characteristic is calculated at the tweet level, and then an aggregation function is applied to the values related to the 100 most recent tweets. For instance, the LENGTH CHARACTERS feature measures the number of characters in each tweet. Then, the 100 values obtained by each account are aggregated using six functions: mean, median, mode, min, max, and variance. In this category of features, we use features that measure the use of different linguistic symbols, such as emoticons, questions, and exclamation marks. Moreover, we include Twitter symbols such as hashtags, mentions, RTs, and replies. We also measure features that are extracted using Spanish-based NLP tools, which are denoted in red. To accomplish this, we used POS and NER tagging algorithms based on the Transformer^50^ pre-trained language model in Spanish, which was provided by Spacy (https://spacy.io/). Finally, we use features that measure sentiment polarities by employing VADER in Spanish (valence, arousal, and dominance scores)^51^.

**Table 1:** List of 265 features used by Botcheck.

	Feature	Description
CONTENT (100 MOST RECENT TWEETS)	LENGTH CHARACTERS	Length of the text, in characters
	LENGTH WORDS	$\ldots$ In number of words
	COUNT UPPERCASE LETTERS	Fraction of capital letters in the tweet
	COUNT EXCLAMATION MARKS	$\ldots$ Exclamation marks in the tweet
	COUNT QUESTION MARKS	$\ldots$ Question marks in the tweet
	COUNT HASHTAGS	$\ldots$ Hashtags in the tweet
	COUNT EMOTICONS	$\ldots$ Emoticons in the tweet
	COUNT RETWEET MARKS	$\ldots$ Retweet marks in the tweet
	COUNT MENTION MARKS	$\ldots$ Mention marks in the tweet
	COUNT URLS	$\ldots$ URLs in the tweet
	COUNT HAPPY EMOJIS	$\ldots$ Happy emojis in the tweet
	COUNT ANGRY EMOJIS	$\ldots$ Angry emojis in the tweet
	COUNT SURPRISE EMOJIS	$\ldots$ Surprise emojis in the tweet
	COUNT SAD EMOJIS	$\ldots$ Sad emojis in the tweet
	COUNT LOVE EMOJIS	$\ldots$ Love emojis in the tweet
	WORD ENTROPY	Shannon entropy of the words in the tweet
	**VALENCE SCORE**	Valence score of the tweet
	**AROUSAL SCORE**	Arousal score of the tweet
	**DOMINANCE SCORE**	Dominance score of the tweet
	**COUNT VB**	Fraction of verbs in the tweet
	**COUNT DT**	$\ldots$ Determiners in the tweet
	**COUNT NOUNS**	$\ldots$ Nouns in the tweet
	**COUNT PROPN**	$\ldots$ Proper nouns in the tweet
	**COUNT ADP**	$\ldots$ Ad-positions in the tweet
	**COUNT PERSONS**	$\ldots$ Persons (entity) in the tweet
	**COUNT LOCATIONS**	$\ldots$ Locations (entity) in the tweet
	**COUNT ORGANIZATIONS**	$\ldots$ Organizations (entity) in the tweet
	**COUNT MISCS**	$\ldots$ Miscellaneous entities in the tweet
TIMELINE	RT RATE	Fraction of retweets in the timeline
	REPLY RATE	Fraction of replies in the timeline
	MENTION RATE	Fraction of mentions in the timeline
	*EXCLAMATION RATE*	$\ldots$ Tweets with exclamation marks in the timeline
	*QUESTION RATE*	$\ldots$ Tweets with question marks in the timeline
	*HASHTAG RATE*	$\ldots$ Tweets with hashtags in the timeline
	*URL RATE*	$\ldots$ Tweets with URLs in the timeline
	*MENTION ENTROPY*	Shannon entropy of the tweets with mentions in the timeline
	*HASHTAG ENTROPY*	Shannon entropy of the tweets with hashtags in the timeline
USER PROFILE	*USERNAME*	Username features: Length in chars, length in words, count of uppercase, excl. marks, question marks, hashtags, emoticons, RT, URLs, emojis (happy, angry, surprise, sad, love), word entropy,** valence, arousal, dominance, POS (VB, DT, NOUNS, PROPN, ADP), NER (PER, LOCS, ORGS, MISC)**
	*NAME*	Name features: Length in chars, length in words, count of uppercase, excl. marks, question marks, hashtags, emoticons, RT, URLs, emojis (happy, angry, surprise, sad, love), word entropy,** valence, arousal, dominance, POS (VB, DT, NOUNS, PROPN, ADP), NER (PER, LOCS, ORGS, MISC)**
	*DESCRIPTION*	Description features: Length in chars, length in words, count of uppercase, excl. marks, question marks, hashtags, emoticons, RT, URLs, emojis (happy, angry, surprise, sad, love), word entropy,** valence, arousal, dominance, POS (VB, DT, NOUNS, PROPN, ADP), NER (PER, LOCS, ORGS, MISC)**
	VERIFIED	Profile verification, ( 1 if is ’true’, 0 otherwise)
	FOLLOWERS COUNT	Number of followers of this user
	FOLLOWING COUNT	Number of friends of this user
	LISTED COUNT	Number of public lists this user is a member of
	TWEET COUNT	Number of tweets authored by this user

Features in black were previously introduced by Botometer^14^. All these features are language-agnostic. Features in bold are computed using Spanish-based NLP tools. Features in italics indicate new features introduced by Botcheck.

We utilize the account timeline as our second source of information. This resource provides us with valuable insight into the patterns of interaction and utilization of each account. To accomplish this, we calculate rates that gauge the use of specific types of interaction over the total number of user posts. We compute rates for RTs, replies, and mentions. Additionally, we have created new features specifically for this source, which we have highlighted in green. These features include rates for exclamations, questions, hashtags, and URLs. Furthermore, we measure the Shannon’s entropy of the words used in posts corresponding to mentions and hashtags.

Finally, we also characterize the user profile, considering the username, name, and description of the account. We perform a linguistic analysis of the profile’s content. It is important to note that unlike the content source (which consists of the 100 most recent tweets), the characteristics of the profile are not distributional since each field is unique. All of these features are new and, accordingly, are highlighted in green. This category also includes metadata such as followers count, following count, listed count, and tweet count.

### Data

Twibot-20^28^ is a dataset that comprises a large corpus of annotated accounts. Twibot was created using crowdsourcing, following annotation guidelines that allowed annotators to mark accounts as bots or humans according to the following criteria: (a) Lack of pertinence and originality in tweets, (b) Highly automated activity and API usage, (c) Tweets containing external links promoting phishing or commercials, and (d) Repeated and redundant tweets (near-duplicates). Twibot-20 only includes accounts with matches in at least 4 out of 5 annotators to eliminate low-agreement annotations. Twibot-20 includes accounts in many languages, of which 6511 are accounts whose contents mostly correspond to Spanish-written tweets. In this corpus partition, there are 2929 bot accounts and 3582 human accounts (i.e., a bot ratio equivalent to 45/55), which were provided to this study by the authors of Twibot-20.

**Figure 2:**
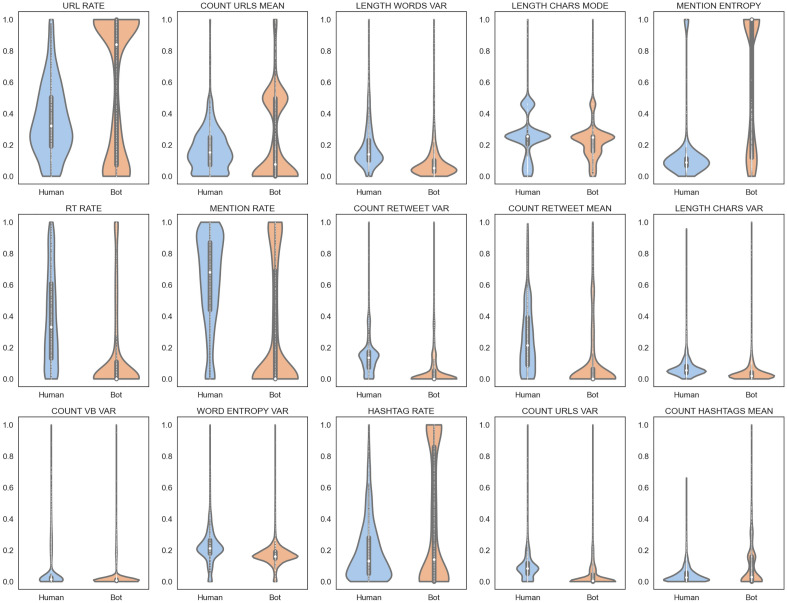
Top-15 most salient features according to information gain. There are differences between bots and humans in the use of hashtags, RTs, URLs, and mentions. We also observe differences in the use of linguistic abstractions expressed in the use of verbs and lexical richness. Finally, there are differences in the length of the messages.

We list the top 15 most relevant characteristics according to information gain. Figure 2 presents violin plots of these characteristics, effectively illustrating the distribution of each variable by combining box plots with kernel density estimation (KDE) graphs.

Figure 2 shows that some features are very informative to distinguish bots and humans. URL RATE shows that a large proportion of bots include URLs in their tweets while humans have a much lower proportion of URLs in their posts. Human tweets are longer than bot tweets, which is evident in both words (LENGTH WORDS VAR) and chars (LENGTH CHARS MODE). Regarding retweets, bots make little use of this type of interaction, which is evident at the timeline (RT RATE) and content (COUNT RETWEET VAR and COUNT RETWEETS MEAN) level. This finding is interesting since bot studies in English show higher use of retweets in bots than in humans, suggesting a major presence of spambots^17^. However, the presence of this type of bot in Twibot-20 (Spanish partition) is scarce.

In terms of linguistic characteristics, humans utilize a broader range of verbs (COUNT VB VAR) compared to bots and exhibit higher lexical diversity (WORD ENTROPY VAR). Finally, bots use more hashtags than humans, evidenced both in the timeline (HASHTAG RATE) and in the content of the messages (COUNT HASHTAG MEAN).

We summarize the main findings made from the exploratory analysis of characteristics in the following list:

- There are differences between bots and humans according to types of interactions. These differences are expressed in asymmetries in the use of hashtags, RTs, URLs, and mentions.
- There are differences between bots and humans in the use of linguistic abstractions. These differences are expressed in asymmetries in the use of verbs and lexical richness.
- There are differences between bots and humans regarding the lengths of the messages.

### Model selection

We evaluate different machine-learning techniques to build Botcheck. We approach a binary classification problem with a class balance ratio of 45/55. To ensure class balance across folds, we employ a training-testing model selection strategy based on stratified 5-fold cross-validation. This approach guarantees that each fold has a roughly equal proportion of samples from each class. This ensures that the model is evaluated on a representative sample of the dataset. Each model was trained to minimize the binary cross-entropy loss.

We study the performance of several binary classification techniques. We used Linear Support Vector Classification (Linear SVC), logistic regression, Random Forest, Multilayer Perceptron (MLP), and AdaBoost. We also explored the use of pre-trained language models for Spanish. We used four different language models: FastText^52^, BETO (https://huggingface.co/dccuchile/bert-base-spanish-wwm-cased), RoBERTa Twitter^32^, and XLM-T^53^. FastText is a pre-trained language model based on subwords that provides word embeddings for Spanish and other languages. BETO is a transformer-based encoder that was trained on a Spanish corpus using the same approach as BERT^54^. RoBERTa Twitter (RoBERTa-tw) is a BERT-based model that modifies key hyperparameters, removing the next-sentence pretraining objective and training with much larger mini-batches and learning rates. RoBERTa-tw was trained using tweets in many languages. XML-T is a model to train and evaluate multilingual language models in Twitter. XLM-T is a cross-lingual language model based on XLM-R, a pretrained language model on 100 languages based on RoBERTa^55^.

For each account, we created a document consisting of their 100 most recent tweets, excluding retweets. We converted each document into a sequence of tokens. We processed these sequences using a maximum number of tokens defined by the longest document in the collection, which had 16,831 tokens. Then, we encoded these sequences into fixed-length inputs of 1,736 tokens, which corresponded to the longest sequence of tokens. We added post-padding to shorter input sequences. Next, we ingested these sequences into the embeddings layer of each language model. The resulting vectors for each document were then combined using a lambda layer. We tested various methods for combining the embeddings, including sums, averages, and concatenations. The best results were obtained by averaging the embeddings. Finally, we used various models to classify the accounts, including 1D convolutional neural networks and feed-forward networks. The feed-forward networks were found to be the most effective.

Table 2 shows performance, on average, in testing folds. The methods show performance across humans (class 0) and bots (class 1).

**Table 2:** Model selection metrics (testing folds) using different machine learning methods.

Method	Accuracy	Slice	Precision	Recall	$F_1$ score
Linear SVC	0.875 ± 0.009	0	0.86 ± 0.006	0.92 ± 0.003	0.89 ± 0.005
		1	0.89 ± 0.011	0.82 ± 0.008	0.86 ± 0.010
		Macro avg	0.88 ± 0.005	0.87 ± 0.003	0.87 ± 0.009
		Micro avg	0.87 ± 0.008	0.88 ± 0.008	0.88 ± 0.008
Logistic regression	0.879 ± 0.006	0	0.86 ± 0.004	0.93 ± 0.004	0.90 ± 0.004
		1	0.91 ± 0.007	0.81 ± 0.005	0.86 ± 0.006
		Macro avg	0.88 ± 0.005	0.87 ± 0.004	0.88 ± 0.005
		Micro avg	0.88 ± 0.004	0.88 ± 0.004	0.88 ± 0.004
Random Forest (n=50)	**0.914** ± 0.003	0	*0.89* ± 0.005	**0.96** ± 0.004	**0.93** ± 0.005
		1	**0.95** ± 0.007	*0.86* ± 0.005	**0.90** ± 0.006
		Macro avg	**0.92** ± 0.004	**0.91** ± 0.003	**0.91** ± 0.004
		Micro avg	**0.92** ± 0.003	**0.92** ± 0.003	**0.92** ± 0.003
MLP ([100, 50], ReLU	*0.902* ± 0.007	0	**0.90** ± 0.006	0.93 ± 0.003	*0.91* ± 0.005
		1	0.91 ± 0.006	**0.87** ± 0.004	*0.89* ± 0.005
		Macro avg	0.90 ± 0.004	*0.90* ± 0.003	*0.90* ± 0.003
		Micro avg	*0.90* ± 0.004	*0.90* ± 0.004	*0.90* ± 0.004
AdaBoost (n=50)	0.886 ± 0.012	0	0.87 ± 0.011	0.93 ± 0.012	0.90 ± 0.011
		1	0.91 ± 0.013	0.84 ± 0.011	0.87 ± 0.012
		Macro avg	0.89 ± 0.011	0.88 ± 0.011	0.88 ± 0.011
		Micro avg	0.88 ± 0.010	0.89 ± 0.009	0.89 ± 0.009
FastText + FFNN	0.897 ± 0.006	0	0.87 ± 0.005	**0.96 ± 0.006**	*0.91* ± 0.005
		1	*0.94* ± 0.004	0.83 ± 0.004	*0.89* ± 0.004
		Macro avg	*0.91* ± 0.005	0.89 ± 0.004	0.89 ± 0.005
		Micro avg	*0.90* ± 0.003	*0.90* ± 0.004	*0.90* ± 0.003
BETO + FFNN	0.852 ± 0.021	0	0.85 ± 0.018	0.89 ± 0.019	0.87 ± 0.018
		1	0.85 ± 0.023	0.81 ± 0.021	0.83 ± 0.022
		Macro avg	0.85 ± 0.020	0.85 ± 0.021	0.85 ± 0.020
		Micro avg	0.85 ± 0.019	0.85 ± 0.020	0.85 ± 0.020
RoBERTa-tw + FFNN	0.867 ± 0.008	0	0.82 ± 0.007	*0.95* ± 0.008	0.88 ± 0.008
		1	0.93 ± 0.006	0.78 ± 0.007	0.85 ± 0.007
		Macro avg	0.88 ± 0.007	0.86 ± 0.006	0.87 ± 0.007
		Micro avg	0.88 ± 0.006	0.87 ± 0.006	0.87 ± 0.006
XLM-T + FFNN	0.874 ± 0.007	0	0.84 ± 0.006	*0.95* ± 0.007	0.89 ± 0.006
		1	0.93 ± 0.003	0.79 ± 0.004	0.85 ± 0.004
		Macro avg	0.89 ± 0.004	0.87 ± 0.004	0.87 ± 0.004
		Micro avg	0.88 ± 0.004	0.87 ± 0.003	0.87 ± 0.004

Bold fonts indicate the best results and italicized fonts the seconds.

Table 2 shows the best results by evaluation metric in bold fonts. We underscore the second-best result. The results show that Random Forest outperforms its competitors. MLP is a very competitive method, obtaining the best precision result in the human class and the best recall result in the bot class. However, Random Forest wins in all other comparisons, achieving the best accuracy and $F_1$ scores. In addition, these results show that Random Forest outperformed FastText, BETO, RoBERTa-tw and XLM-T, corroborating other studies that have also found that Spanish-pre-trained language models fail to overcome ML methods in text classification^56^. Accordingly, we select Random Forest to classify bots and humans with Botcheck.

### Ablation study

We conduct an ablation study to assess the influence of various feature sets in Botcheck. We evaluate the performance of Botcheck versions that omit certain features: those based on Botometer features (indicated in black), Spanish-based NLP features (red), and novel features (green). We also analyze the performance degradation when each feature source is excluded, specifically focusing on content, timeline, and profile sources. Lastly, we train versions of Botcheck restricted to using only one of these sources at a time, resulting in Botcheck versions exclusively based on content, timeline, or profile features. The outcomes of this analysis are presented in Table 3.

**Table 3:** Ablation study obtained by removing different partitions of features.

Method	Accuracy	Slice	Precision	Recall	$F_1$ score
Botcheck${\setminus }$Botometer features (black)	0.867 ± 0.003	0	0.84 ± 0.004	0.94 ± 0.004	0.89 ± 0.003
		1	0.91 ± 0.003	0.78 ± 0.004	0.84 ± 0.003
		Macro avg	0.88 ± 0.003	0.86 ± 0.004	0.86 ± 0.003
		Micro avg	0.87 ± 0.003	0.87 ± 0.003	0.87 ± 0.003
Botcheck${\setminus }$Spanish NLP features (red)	0.883 ± 0.008	0	*0.86* ± 0.007	*0.95* ± 0.006	*0.90* ± 0.006
		1	0.92 ± 0.010	0.81 ± 0.011	0.86 ± 0.010
		Macro avg	*0.89* ± 0.008	*0.88* ± 0.007	*0.88* ± 0.008
		Micro avg	*0.89* ± 0.007	0.88 ± 0.007	0.88 ± 0.007
Botcheck${\setminus }$novel features (green)	0.882 ± 0.012	0	*0.86* ± 0.011	0.94 ± 0.010	*0.90* ± 0.011
		1	0.92 ± 0.009	0.81 ± 0.010	0.86 ± 0.010
		Macro avg	*0.89* ± 0.010	*0.88* ± 0.009	*0.88* ± 0.010
		Micro avg	0.88 ± 0.010	0.88 ± 0.010	0.88 ± 0.010
Botcheck${\setminus }$content features	0.816 ± 0.010	0	0.78 ± 0.008	0.92 ± 0.007	0.85 ± 0.008
		1	0.88 ± 0.012	0.69 ± 0.011	0.77 ± 0.012
		Macro avg	0.83 ± 0.010	0.80 ± 0.009	0.81 ± 0.009
		Micro avg	0.83 ± 0.010	0.82 ± 0.009	0.81 ± 0.010
Botcheck${\setminus }$timeline features	0.885 ± 0.005	0	*0.86* ± 0.006	0.94 ± 0.005	*0.90* ± 0.006
		1	0.92 ± 0.002	*0.82* ± 0.002	*0.87* ± 0.002
		Macro avg	*0.89* ± 0.004	*0.88* ± 0.005	*0.88* ± 0.004
		Micro avg	*0.89* ± 0.004	*0.89* ± 0.004	*0.89* ± 0.004
Botcheck${\setminus }$user profile features	0.883 ± 0.006	0	*0.86* ± 0.005	0.94 ± 0.004	*0.90* ± 0.004
		1	0.92 ± 0.004	*0.82* ± 0.004	0.86 ± 0.005
		Macro avg	*0.89* ± 0.005	*0.88* ± 0.007	*0.88* ± 0.006
		Micro avg	*0.89* ± 0.005	0.88 ± 0.005	0.88 ± 0.005
Botcheck [content features]	0.886 ± 0.012	0	*0.86* ± 0.011	*0.95* ± 0.012	*0.90* ± 0.012
		1	*0.93* ± 0.010	0.81 ± 0.012	*0.87* ± 0.011
		Macro avg	*0.89* ± 0.010	*0.88* ± 0.010	*0.88* ± 0.010
		Micro avg	*0.89* ± 0.009	*0.89* ± 0.009	*0.89* ± 0.009
Botcheck [timeline features]	0.801 ± 0.015	0	0.78 ± 0.018	0.88 ± 0.012	0.83 ± 0.016
		1	0.83 ± 0.011	0.70 ± 0.012	0.76 ± 0.012
		Macro avg	0.81 ± 0.012	0.79 ± 0.013	0.79 ± 0.012
		Micro avg	0.80 ± 0.011	0.80 ± 0.011	0.80 ± 0.011
Botcheck [user profile features]	0.543 ± 0.018	0	0.55 ± 0.016	**0.96 ± 0.016**	0.70 ± 0.017
		1	0.33 ± 0.023	0.01 ± 0.017	0.02 ± 0.019
		Macro avg	0.44 ± 0.020	0.50 ± 0.017	0.36 ± 0.018
		Micro avg	0.45 ± 0.019	0.54 ± 0.019	0.40 ± 0.019
Botcheck (Best model—all features)	**0.914 ± 0.003**	0	**0.89 ± 0.005**	**0.96 ± 0.004**	**0.93 ± 0.005**
		1	**0.95 ± 0.007**	**0.86 ± 0.005**	**0.90 ± 0.006**
		Macro avg	**0.92 ± 0.004**	**0.91 ± 0.003**	**0.91 ± 0.004**
		Micro avg	**0.91 ± 0.003**	**0.93 ± 0.003**	**0.92 ± 0.003**

Significant values are given in bold and italics.

The ablation study demonstrates that the features to which Botcheck is most sensitive are those based on content. Removing these features results in the most significant performance deterioration. Consequently, the model limited to using only content features shows the best performance in the evaluation. Botcheck’s reliance on this type of feature means that eliminating Botometer-based features leads to a decline in performance. Regarding timeline-based features, they perform well, proving more useful than the model relying solely on profile features.

### Inspecting Botcheck

**Figure 3:**
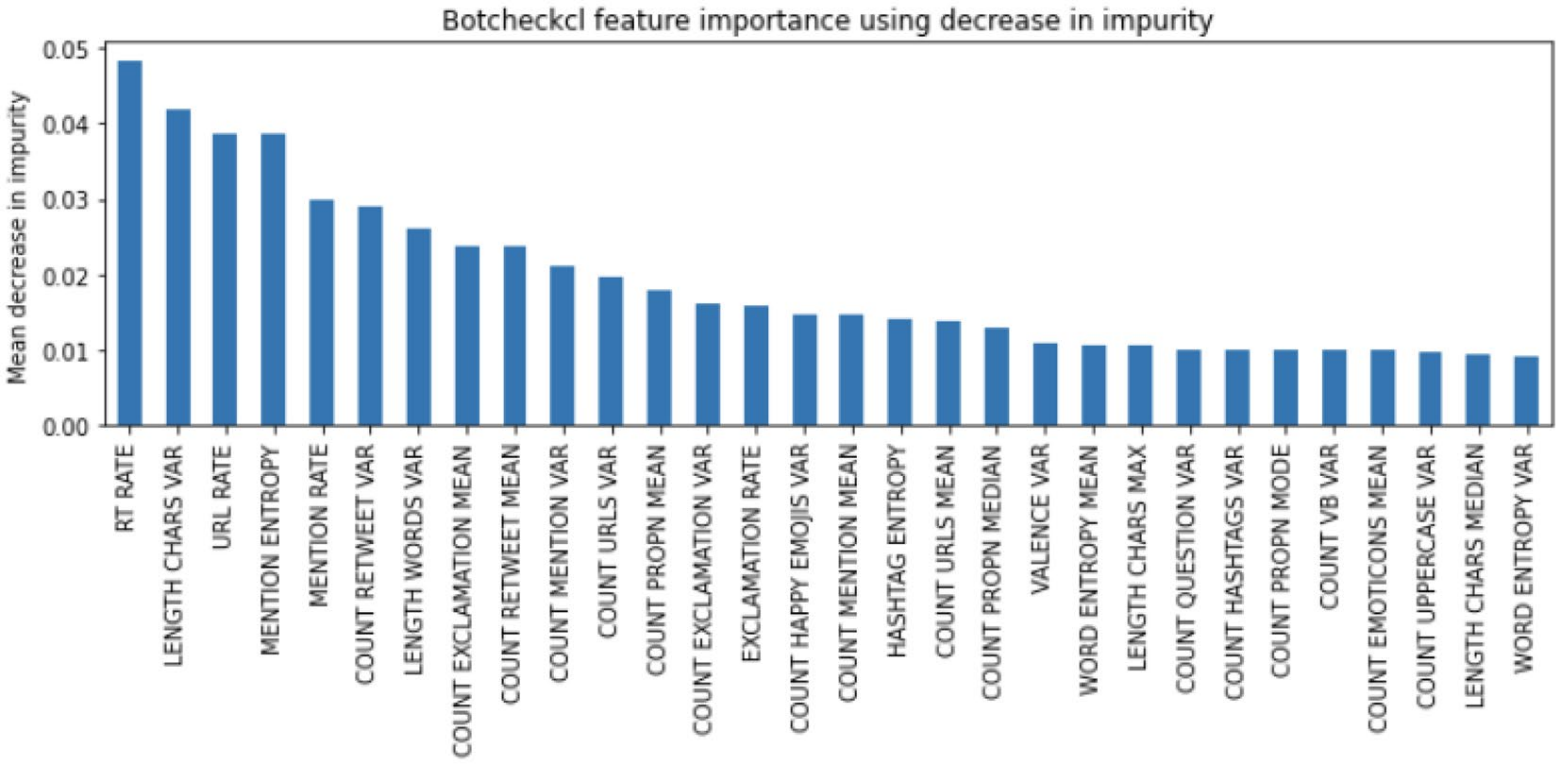
Most salient features according to decrease in impurity.

To understand how Botcheck works, we explored the main features that our detector pays attention to. We use feature importance techniques to highlight essential features of the model. We start elucidating feature importance using the decrease in impurity, traversing all the trees in Botcheck’s Random Forest. The most important features are those that, on average, achieve a higher impurity decrease in the model. Figure 3 shows the top 30 most salient features of Botcheck according to this criteria.

The timeline features prove to be very relevant to Botcheck. RT RATE, URL RATE, and MENTION RATE are among the five most relevant features of the model. This finding coincides with the results shown in Fig. 2, in which the most relevant features are identified using information gain. The content features are also relevant, highlighting LENGTH CHARS VAR and MENTION ENTROPY.

**Figure 4:**
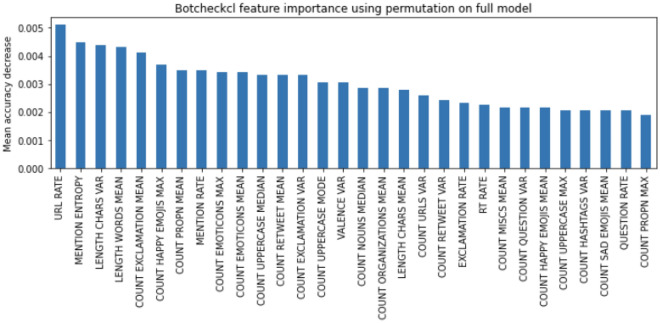
Most salient features according to feature permutation.

One limitation of impurity for feature importance is that the features with greater cardinality have a more significant presence in the analysis since they produce more bisections in the trees and, therefore, may appear more times in the impurity decrease computation. We use a second feature importance criterion to address this limitation, called permutations-based feature importance. To do this, a feature of the validation set is permuted, and the classification accuracy is re-evaluated. Permutation importance is defined as the difference between the base model and the outcome obtained from permuting the feature. Figure 4 shows the top 30 features according to permutation importance.

Figure 4 shows some differences in the feature set relevant to Botcheck. When using permutation importance, the stylometry-based features acquire more relevance than the features extracted from the timeline. For example, MENTION ENTROPY, LENGTH CHARS VAR, LENGTH WORDS MEAN, and COUNT EXCLAMATION MEAN appear within the top-5 most salient features of Botcheck. Of the timeline features, only the RATE URL remains within the top features. Features based on emojis and emoticons also acquire relevance. This analysis reinforces the idea that stylometry reveals salient clues to detect bots. This aligns with the significance of the content features identified in the ablation study.

### A local explainer for Botcheck

**Figure 5:**
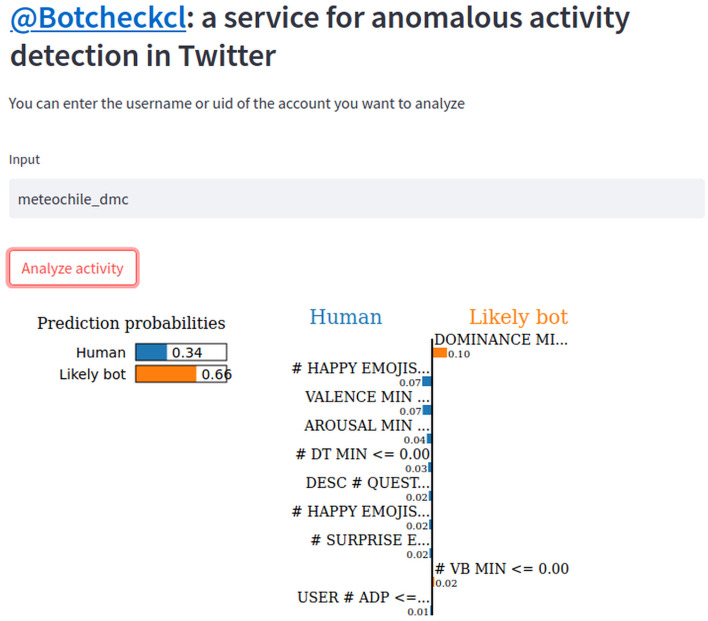
Botcheck provides an explanation that allows distinguishing which account features are more relevant for the model. In the example, we show the report for @meteochile_dmc, the weather report bot of the Chilean Meteorological Agency.

An important feature of Botcheck is that it operates on descriptive features of user-profiles and account usage patterns. These types of features make Botcheck a model that can be explainable. We integrated a local explainer named LIME^57^ to provide explanations for each model output. In this way, Botcheck not only provides the confidence of the majority class, but also shows the most relevant features that allow for the explanation of the model output for each specific example. Figure 5 shows the output produced by Botcheck for the account @meteochile_dmc, the weather report bot of the Chilean Meteorological Agency.

The application of LIME in Botcheck’s report provides evidence supporting the detection of ’likely bots’. Botcheck’s role about these accounts is to notify Twitter, supplying proof of bot activity, including the information from the explainer.

## Experimental results

### Competitive methods

We begin by comparing our Botcheck system with Botometer V.4 ^14^, which serves as the baseline for comparison since our selection of features is derived from Botometer. Initially, we obtained Botometer scores for every account in our dataset. Subsequently, we evaluated the binary classification using Botometer’s Ensemble of Specialized Classifiers (ESC). We used two different versions for this evaluation. The first version does not impose any restrictions on the bot score, assigning the account to the class that receives the highest score. The second version utilizes the Complete Automation Probability (CAP) constraint, which is the likelihood, based on Botometer’s models, that an account with a particular score or above is automated. CAP is utilized to estimate the overall bot prevalence, aiming to achieve a balance between the rates of false positives and false negatives. Following Botometer’s guidelines, we set a CAP of 90%, which is anticipated to produce an approximate false positive rate of 10%.

In addition, to study Botcheck, we compare its performance with that of other competitive methods. Twibot-20^28^ releases user IDs so using the Twitter API we can retrieve metadata from the user profile and the timeline of recent tweets. These data sources, which Botcheck uses, allow evaluating the performance of Infoshield^26^, the one-class classifiers introduced by^33^ and the Random Forest classifier introduced by^27^. Regarding bot detection initiatives in Spanish, Twibot-20 favors the evaluation of the winning method of PAN AP 2019, based on char + word n-grams^40^, and the Isolation Forest classifier proposed by ^45^, based on anomaly detection. In addition, we have included two text classifiers based on language models (BERT and RoBERTa), utilizing tweets that have been translated from Spanish to English. We indicate specific details of each of these methods:

- Botometer^14^: Botometer V.4, accessed through its professional API (https://rapidapi.com/OSoMe/api/botometer-pro). We employ both the universal overall CAP version of Botometer as well as the universal overall unconstrained version of the method.
- BERT (large)^54^: A State-of-the-Art pretrained text model applied to tweets translated from Spanish to English Using Google Translate API. Classification is performed Using Feed-Forward Neural Networks (FFNN).
- RoBERTa^32^: A State-of-the-Art pretrained text model applied to tweets translated from Spanish to English Using Google Translate API. Classification is performed Using Feed-Forward Neural Networks (FFNN).
- Infoshield^26^: we evaluate the original implementation of Infoshield, which can find language-independent clusters of bots in Twitter (https://github.com/mengchillee/Infoshield).
- RF^27^: the Random Forest classifier that is based on user metadata and derived secondary features. This method is language agnostic, favoring its evaluation in this task. This method achieves the best results in Twibot-20 according to the experiments developed by^28^.
- BRM^33^:^33^ evaluated many one-class classifiers in this task, including one-class SVM and Bagging-Random Miner (BRM). We include BRM^58^, a one-class classifier ensemble that achieves competitive results in the task. The authors provided an implementation of BRM (https://github.com/miguelmedinaperez/Bagging-RandomMiner).
- Char + word n-grams^40^: we evaluate the char + word n-grams method that won the PAN AP 2019 contest. We reimplemented this method following the specifications provided by^40^. Accordingly, we used a tweet tokenizer, replacing URLs and mentions with special tokens. Then, we detokenize the list of tokens using Treebank Word Detokenizer. For word and char vectorization, we used Tf-Idf, with sublinear Tf. For word processing, we used n-grams in {1, 2}, with a minimum frequency threshold = 2. For char processing, we used n-grams in {3, 5}, with a minimum frequency threshold = 2. We used SVC for classification.
- Isolation forest^45^: we used the isolation forest classifier based on the features computed by ^45^. Isolation forest is an anomaly detection method that learns patterns from the background class (humans) and uses a threshold to separate anomalous accounts from this background. The authors fixed the threshold at 7.5%, following findings about the expected volume of bots in Twitter provided by^25^.

### Results

We use stratified 5-fold cross-validation to compare the performance of the studied methods. We report the results per class (0: human, 1: bot). In addition, we also report aggregated results using $F_1$ scores at micro and macro levels. The reported results correspond to average performance metrics computed on testing folds. We show these results in Table 4.

**Table 4:** Performance metrics of competitive methods in Twibot-20 (Spanish partition).

Method	Accuracy	Slice	Precision	Recall	$F_1$ score
Botometer (universal overall CAP)^14^	0.733 ± 0.025	0	0.69 ± 0.028	0.92 ± 0.027	0.79 ± 0.027
		1	0.83 ± 0.026	0.49 ± 0.027	0.62 ± 0.026
		Macro avg	0.76 ± 0.026	0.71 ± 0.026	0.71 ± 0.026
		Micro avg	0.73 ± 0.025	0.73 ± 0.025	0.73 ± 0.025
Botometer (universal overall unconstrained)^14^	0.689 ± 0.029	0	0.71 ± 0.028	0.76 ± 0.032	0.73 ± 0.029
		1	0.66 ± 0.027	0.61 ± 0.022	0.63 ± 0.023
		Macro avg	0.68 ± 0.027	0.68 ± 0.024	0.68 ± 0.024
		Micro avg	0.68 ± 0.025	0.68 ± 0.025	0.68 ± 0.025
BERT^54^	0.847 ± 0.010	0	0.84 ± 0.009	0.89 ± 0.008	0.86 ± 0.009
		1	0.85 ± 0.010	*0.80* ± 0.011^∗^	*0.83* ± 0.010^∗^
		Macro avg	0.85 ± 0.010	0.84 ± 0.009	*0.85* ± 0.010^∗^
		Micro avg	0.85 ± 0.010	0.85 ± 0.010	0.85 ± 0.010
RoBERTa^32^	*0.868* ± 0.009^∗^	0	0.84 ± 0.008	*0.94* ± 0.009	0.89 ± 0.008
		1	*0.92* ± 0.009	0.78 ± 0.008	*0.84* ± 0.008^∗^
		Macro avg	*0.88* ± 0.009^∗^	0.86 ± 0.009	*0.86* ± 0.009^∗^
		Micro avg	*0.87* ± 0.008^∗^	0.87 ± 0.008	*0.87* ± 0.008^∗^
Infoshield^26^	0.852 ± 0.008	0	0.86 ± 0.009	0.83 ± 0.008	0.84 ± 0.008
		1	0.84 ± 0.009	0.74 ± 0.009	0.79 ± 0.009
		Macro avg	0.85 ± 0.009	0.79 ± 0.009	0.82 ± 0.009
		Micro avg	0.85 ± 0.008	0.80 ± 0.008	0.82 ± 0.008
RF^27^	*0.868* ± 0.004^∗^	0	0.86 ± 0.004	0.89 ± 0.005	0.87 ± 0.004
		1	0.87 ± 0.005	0.71 ± 0.006	0.78 ± 0.005
		Macro avg	*0.87* ± 0.004^∗^	0.80 ± 0.005	0.83 ± 0.004
		Micro avg	*0.86* ± 0.004^∗^	0.83 ± 0.004	0.84 ± 0.004
BRM^33^	0.851 ± 0.011	0	**0.94** ± 0.009	**0.96** ± 0.012^∗^	**0.95** ± 0.010^∗^
		1	0.72 ± 0.013	0.71 ± 0.012	0.71 ± 0.012
		Macro avg	0.83 ± 0.009	0.84 ± 0.010	0.83 ± 0.010
		Micro avg	*0.86* ± 0.008^∗^	0.88 ± 0.009	*0.87* ± 0.008^∗^
Chars + word n-grams^40^	0.848 ± 0.008	0	0.86 ± 0.009	**0.96** ± 0.009^∗^	0.91 ± 0.008
		1	0.81 ± 0.007	*0.81* ± 0.006^∗^	0.81 ± 0.006
		Macro avg	0.84 ± 0.008	*0.89* ± 0.007	*0.86* ± 0.007^∗^
		Micro avg	0.84 ± 0.007	*0.91* ± 0.007	*0.88* ± 0.007^∗^
Isolation Forest^45^	*0.872* ± 0.011^∗^	0	*0.92* ± 0.010	**0.97** ± 0.011^∗^	**0.94** ± 0.010^∗^
		1	0.74 ± 0.012	0.71 ± 0.011	0.72 ± 0.011
		Macro avg	0.83 ± 0.010	0.84 ± 0.011	0.83 ± 0.010
		Micro avg	*0.86* ± 0.009^∗^	0.88 ± 0.010	*0.87* ± 0.010^∗^
Botcheck	**0.914** ± 0.003	0	0.89 ± 0.005	**0.96** ± 0.004^∗^	*0.93* ± 0.005
		1	**0.95** ± 0.007	**0.86** ± 0.005	**0.90** ± 0.006
		Macro avg	**0.92** ± 0.004	**0.91** ± 0.003	**0.91** ± 0.004
		Micro avg	**0.91** ± 0.003	**0.93** ± 0.003	**0.92** ± 0.003

Bold fonts indicate the best results and italicized fonts the seconds. The results marked with ^∗^ indicate that there are no significant differences between the results.

The findings presented in Table 4 highlight several key points. When comparing the two versions of Botometer, it’s observed that the universal unconstrained approach enhances precision in identifying humans and significantly increases recall for bots. In contrast, the method based on CAP control improves precision for bots but reduces recall. Nonetheless, the overall performance using CAP, both at the micro and macro levels, surpasses that of both methods. Additionally, the $F_1$ scores achieved with CAP surpass the other baseline, due to the balance between false positives and negatives achieved with this version of Botometer V.4. However, the performance of both baseline versions falls short compared to the other methods evaluated. Among these, BERT-based methods stand out, ranking second in several evaluations with competitive results in accuracy and $F_1$ scores. Specifically, RoBERTa excels in precision, securing the second position for bot classification at both the micro and macro levels. Its performance in the human class, particularly in recall, is also remarkable, reaching the second position. Another competitive method is RF, which ranks second in accuracy and shows good precision at both the micro and macro levels. Methods vying for the top spots include BRM, with strong performances in the human class; Chars + words n-grams, excelling in human recall; and Isolation Forest, also performing well in human recall and $F_1$ score. These three methods also achieve second places in various evaluations, especially Chars + word n-grams in recall for both classes. Overall, the most solid results come from Botcheck, which achieves the best performance in accuracy and tops the evaluations in the bot class, leading to the best results at both the micro and macro levels across all metrics. Its ability to classify humans demonstrates slightly lower precision than the most competitive methods but still achieves the highest recall along with BRM, Chars + word n-grams, and Isolation Forest.

Botcheck’s success in the bot class is partly due to its binary classification approach, which is effective since Twibot-20 includes a significant number of annotated bot accounts (45%), mitigating severe class imbalance issues. In this context, methods based on one-class classification like BRM and Isolation Forest are less competitive at detecting bots, as reflected in their decreased precision in this class. On the other hand, RoBERTa shows strong performance in this class but at the expense of less competitive recall. Some interesting observations were made regarding RF. In Feng’s experiments (Twibot-20), RF demonstrated solid performance, with an $F_1$ score of 0.85. Our study confirms its effectiveness in the Spanish-based Twibot-20 partition, achieving a comparable $F_1$ score (0.83 at the macro level). Char + word n-grams, relying solely on text-based features, also show good results ($F_1$ score = 0.86 at the macro level), highlighting the importance of such features in this task. Botcheck’s advantage comes from leveraging both text and user-based features, explaining its performance.

## Case study: 2021 Chilean presidential elections

We present an analysis of bot activity from the Chilean 2021 presidential campaign on Twitter. These elections are an interesting case study because none of the candidates of traditional center-right and center-left were successful in the first round, leaving two candidates, one aligned with a progressive left (Gabriel Boric) and another with the conservative right (José Antonio Kast), to run for the presidency.

The study case is highly significant as it allows us to evaluate Botcheck in a different context (the 2021 political campaign in Chile) compared to the environment prevalent during the collection and annotation of accounts in Twibot-20 (Twitter accounts up to the year 2020). There are two key aspects in this study: 1) Assessing the performance of Botcheck in a transfer learning scenario, based on the comparison of accounts detected by Botcheck against annotations made by a group of human annotators, and 2) Analyzing the implications of bot detection in the context of a political campaign. The analysis addresses the political leanings of the bot accounts and correlates them with affective polarization.

We start by analyzing all the Twitter accounts that follow the official candidates’ Twitter accounts @gabrielboric and @joseantoniokast. The massive collection of accounts involved the analysis of approximately 28,000 accounts per day, a significant amount of accounts considering the upper limit of requests allowed by the API (86,400). In 37 days (November 1, 2021, to December 9, 2021), the population reached 1,060,509 accounts. In Boric’s case, Twitter recorded 591,402 followers, and in Kast’s, 469,107 followers. We selected a sample from this population, using a 10% sampling rate (106,050 accounts). This sample size ensured that we did not exceed our monthly download limit of 10 million tweets. For the sampling process, we employed stratified sampling, preserving the proportion of followers of each account.

To validate the effectiveness of Botcheck in this case study, we took a random sample of 1% of the total number of accounts analyzed by Botcheck, corresponding to 1,060 accounts. These accounts were analyzed by a group of annotators at our research center.

To annotate these accounts, they followed the annotation guidelines used by Twibot-20, with five annotations per account, keeping the accounts where the agreement involves at least four annotators. As a result, the data consider 875 annotated accounts, of which 71 were annotated as bots and 804 as humans, with an 8% proportion of bots in the sample. Of the accounts on which the annotators did not have agreement (185), 52 accounts were not accessible during the experiment. Out of the 71 bots annotated by humans, Botcheck identified 67 as bots and 4 as humans. Conversely, of the 804 accounts labeled as humans by annotators, 784 were classified as humans and 20 as bots by Botcheck. We used these data to assess Botcheck’s performance in a transfer learning scenario. These results are presented in Table 5.

**Table 5:** Performance of Botcheck on human-annotated data during the case study.

Slice	Precision	Recall	$F_1$ score
0	0.99	0.98	0.99
1	0.77	0.94	0.85
Macro avg	0.88	0.96	0.92
Micro avg	0.97	0.97	0.97

Table 5 presents the effectiveness of Botcheck, as measured against the annotated data from the case study. The results evaluate Botcheck’s performance in a scenario of transfer learning. This case study used data from accounts annotated in 2021, differing from the data in Twibot-20, which includes accounts annotated before 2020. This difference highlights a key observation: Botcheck’s precision in detecting bots diminishes over time, even though it continues to demonstrate a high rate of correctly identifying bots (high recall). Notably, Botcheck shows almost perfect performance in identifying human accounts (class 0), a result typically seen when the method is adjusted to increase recall for detecting bots (the target class). An important aspect of Botcheck is its capability to accurately identify almost all bots recognized by human annotators. However, this comes at the cost of overestimating the bot class.

**Figure 6:**
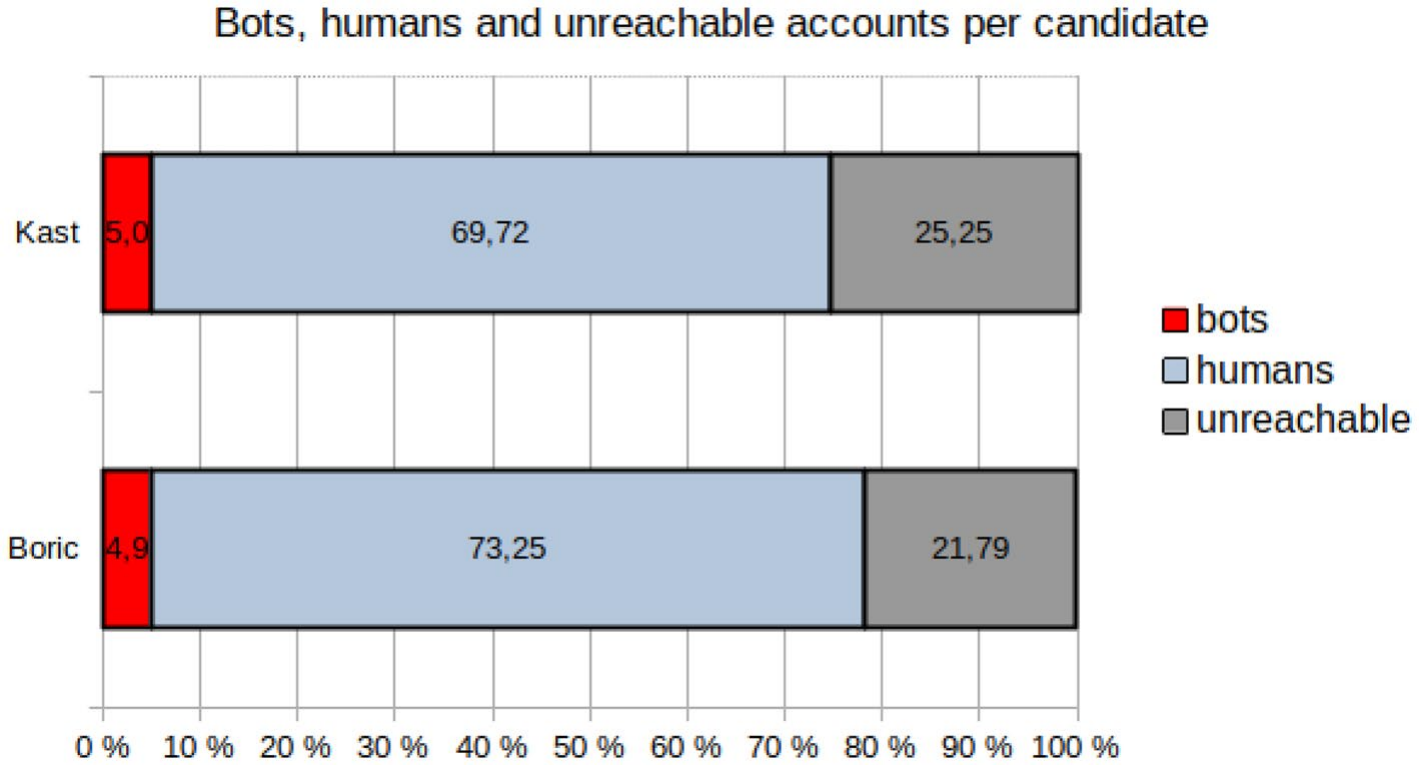
Bots, humans, and unreachable accounts per candidate.

Then, we completed the checking of accounts in the sample totaling 106,050 accounts, with 56,030 accounts that follow Boric, 42,804 accounts that follow Kast, and 7,216 that follow both. After running Botcheck on the sample, we detected a majority of humans and a small fraction of bots (5,038 accounts). Figure 6 shows that approximately 5% of the accounts that follow each candidate were classified as bots (2,142 accounts for Kast, 2,691 accounts for Boric, and 205 accounts that follow both candidates). However, the analysis also reveals a very high volume of unreachable accounts. These are accounts that appear in each candidate’s list of followers but are not accessible through the API. Unreachable accounts could be related to detecting inauthentic activity by Twitter (such as bots) or the violation of the platform’s terms of use by hate speech and harassment.

We constructed a collection of tweets issued by bots detected by Botcheck in the case study (5,038 accounts). These tweets were retrieved by Botcheck by inferring the account type, utilizing the 100 most recent tweets obtained from the API. We were unable to recover 100 tweets from every account, as some were new and had lesser activity. We removed accounts that made less than 10 tweets (37 accounts). In total, the collection consists of 368,831 tweets, with an average of 73.75 tweets per analyzed account (5001 accounts). Then, we inspected the timeline of some accounts and observed two clear emerging patterns. Some bots support or amplify the candidate’s message but others also attack the opponent’s account. For this reason, we determined that it was essential for the study to analyze the content transmitted by bots, for example, in terms of political orientation and affective polarization. By estimating the political position of the bots, we can determine how aligned they are with the ideological orientation of each candidate. Furthermore, we can infer to some extent their intent by determining whether bot messages have a positive or negative emotional polarization when interacting with each of the candidates: those aligned with their ideology and those on the opposite pole. To comprehend the role that bots play among the analyzed accounts, we also conducted an analysis on accounts labeled as humans by Botcheck.

**Figure 7:**
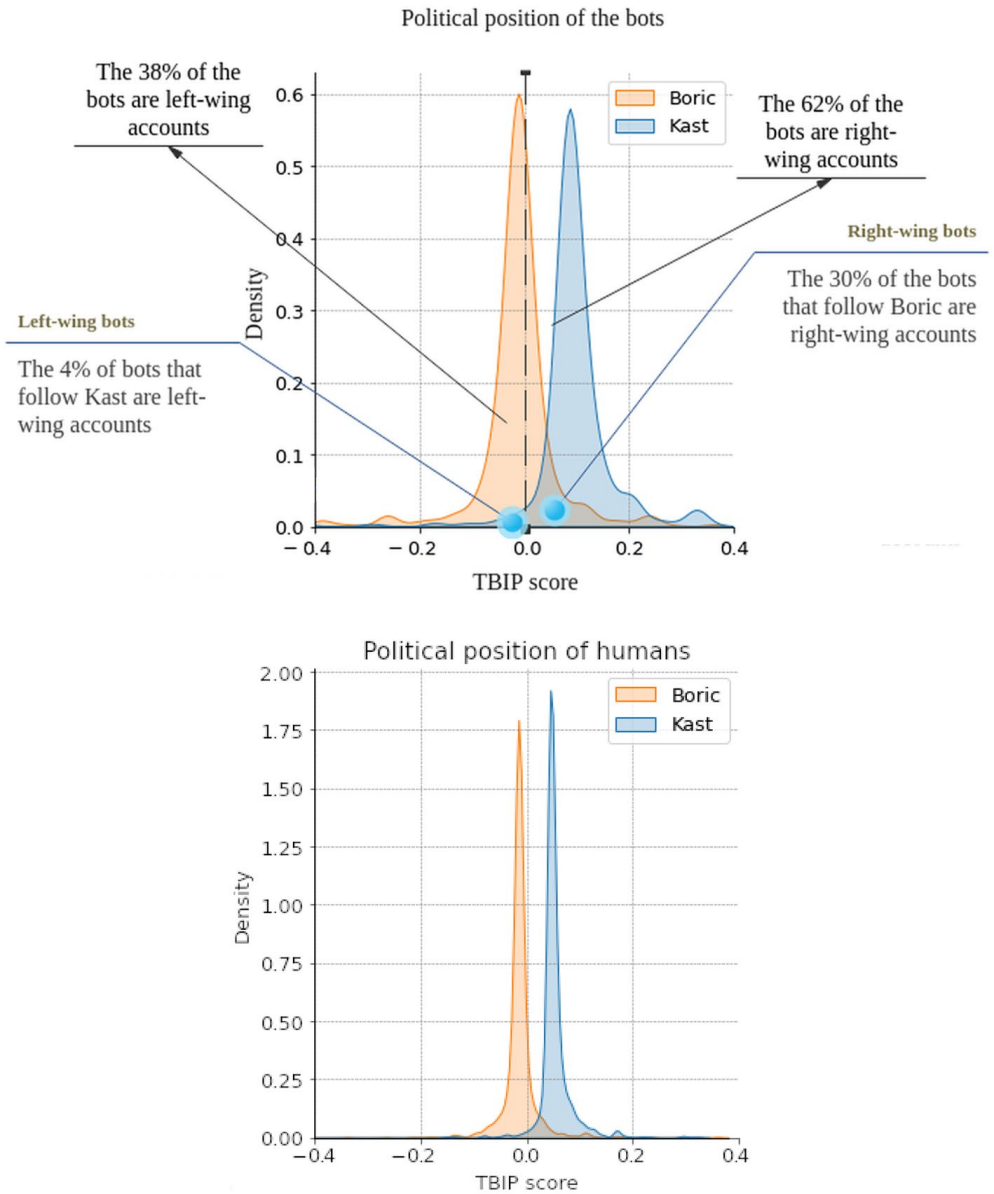
Political orientation of bots (top) and humans (bottom) (density plots).

To infer the political orientation of each bot account, we used Text-based Ideal Points (TBIP)^59^, a method that analyzes the messages of the accounts and assigns a score on the left-right axis. TBIP is an unsupervised algorithm based on probabilistic topic models that analyze texts to quantify the political position of users of a social network. One of the advantages of TBIP is that it can estimate the political position of any user who writes contingent texts. We used the corpus of 368,831 tweets to infer the TBIP score of each of the accounts classified as bots. We employed the same procedure recommended in the TBIP documentation to process the corpus. Accordingly, we removed stopwords. We include all unigrams, bigrams, and trigrams that appear in at least 0.1% of tweets. To train TBIP, we perform stochastic gradient ascent using Adam with a mini-batch size of 512. We use a single Monte Carlo sample to approximate the gradient of each batch. We assume 50 latent topics and posit Gamma(0.3, 0.3) and N(0, 1) for priors. After adjusting the model based on bots, we performed inference on the political positions of human accounts using the pre-trained model on bots. This approach allows us to understand the relative position of human accounts concerning the spectrum identified using the bots’ tweets, determining whether there are differences or if they correspond to scores sampled from the same distributions.. Figure 7 shows the distribution of bots and humans per candidate along the continuous left-right orientation axis calculated using TBIP.

This exercise reveals asymmetries between the bots following Boric and Kast. According to the political spectrum identified by TBIP, bots with a right-wing ideological orientation predominate, reaching 62% of the total, while those with a left orientation represent 38%. Furthermore, the bots that follow Boric are mostly center or center-left (close to 0 on the horizontal axis). In contrast, the bots that follow Kast are on the right (close to 1 on the horizontal axis). Even though not all bots are ideologically aligned with each candidate, we can affirm that 30% of the bots following Boric are ideologically opposed to him, while only 4% of those following Kast are. Regarding the bots that fall at the extremes of the distributions, we have calculated the standard deviation for each curve. The points that fall outside the range of $-2\sigma$ to +2$\sigma$ correspond to 2% of the bots that follow Boric and 8% of the bots that follow Kast.

Regarding the political leanings of humans, the figure indicates that the support for the distributions is smaller than that observed with bots. As revealed by the mean test (unpaired T-test), no statistically significant differences were found between the distributions of bots and humans who follow Boric (mean for bots = − 0.015, standard deviation = 0.11; mean for humans = − 0.012, standard deviation = 0.03, p-value = 0.1156). However, a significant difference was detected between the distributions of bots and humans who follow Kast (mean for bots = 0.12, standard deviation = 0.08; mean for humans = 0.06, standard deviation = 0.03, p-value = 0.0001).

Another way to analyze the contents of the bots is to infer the emotional charge of the words they use. To do this, we calculate the linguistic valence of the timeline of each bot account. We estimate an emotional language level according to a like-dislike scale. This technique lets us infer whether a bot account’s messages that follow a candidate have a positive or negative emotional charge.

To measure valence, we used the Spanish version of Affective Norms for English Words^60^, widely known as ANEW, a set of normative emotion scores for more than a thousand words in English. ANEW indicates psycho-linguistic dimensions of emotional charge based on the conceptual framework established by^61^. The first two dimensions are valence on the axis like-dislike and stimulus on the passive-active axis. The third dimension, domain, less related to the first two, establishes the individual’s level of control or self-regulation. Normed verbal markers establish the load on each of these dimensions. For this study, the most relevant dimension is the affective charge of valence since it accounts for the emotional charge of the author of the analyzed text on the axis of liking–disliking. The Spanish version of ANEW was developed by^51^. The Spanish ANEW standards were established using data from 720 participants, comprising 560 female and 160 male psychology students aged 18–25 years. During the 2003–2004 and 2004–2005 academic years, these students evaluated words at various psychology faculties (or equivalent departments) across several Spanish universities. They assessed the Spanish translations of the 1034 words from ANEW. A professional philologist with expertise in English conducted the initial translation, which was then refined by a professor of English philology and a psycholinguistics researcher, under the guidance of the study’s authors. The Spanish version of ANEW has proven particularly useful for investigating how attentional resources are allocated to processing emotional words, depending on their position in the affective space, making it a valuable tool for studying affective polarization^62^.

To calculate the valence scores for each account, we summed up all the scores of words used in the tweets from the analyzed bot accounts that matched those included in the Spanish ANEW. After retrieving these scores, we computed the mean score for each account.

**Figure 8:**
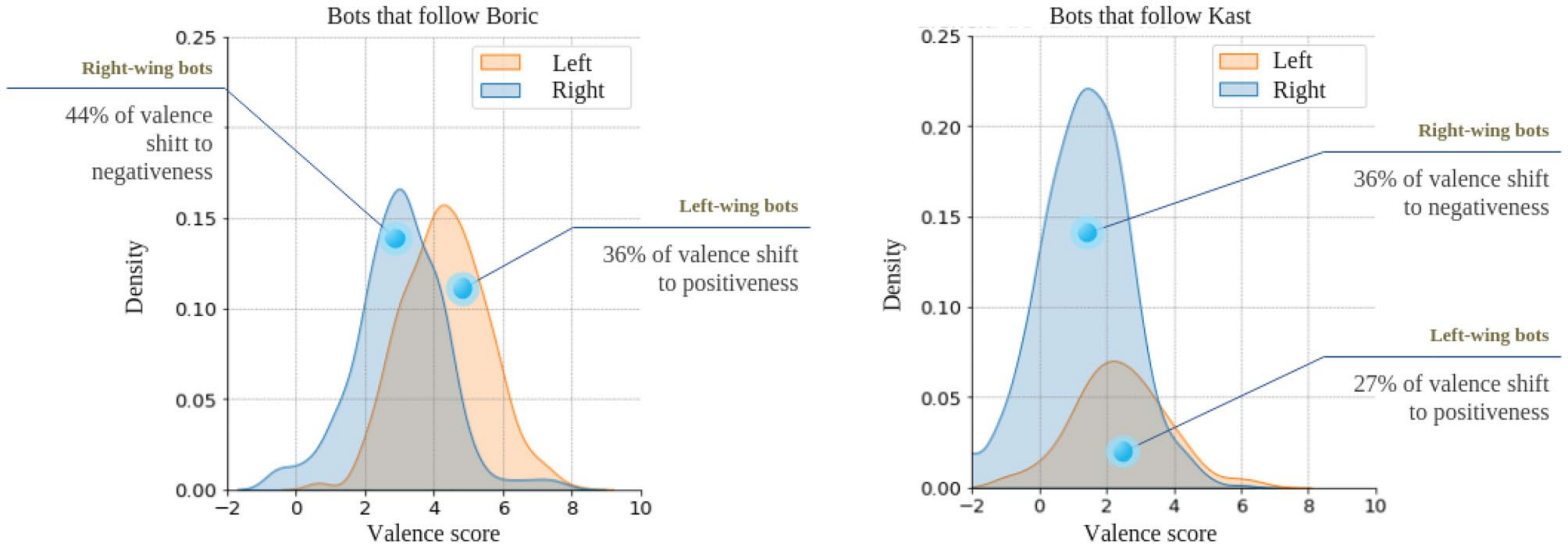
Valence scores (mean) per account (density plots). (Left) Bots that follow Gabriel Boric, and (Right) Bots that follow José Antonio Kast. Valence shift was computed using differences of areas. For example, a valence shift to positiveness shows the difference between two distributions toward positive values, while a valence shift to negativeness shows the opposite.

**Figure 9:**
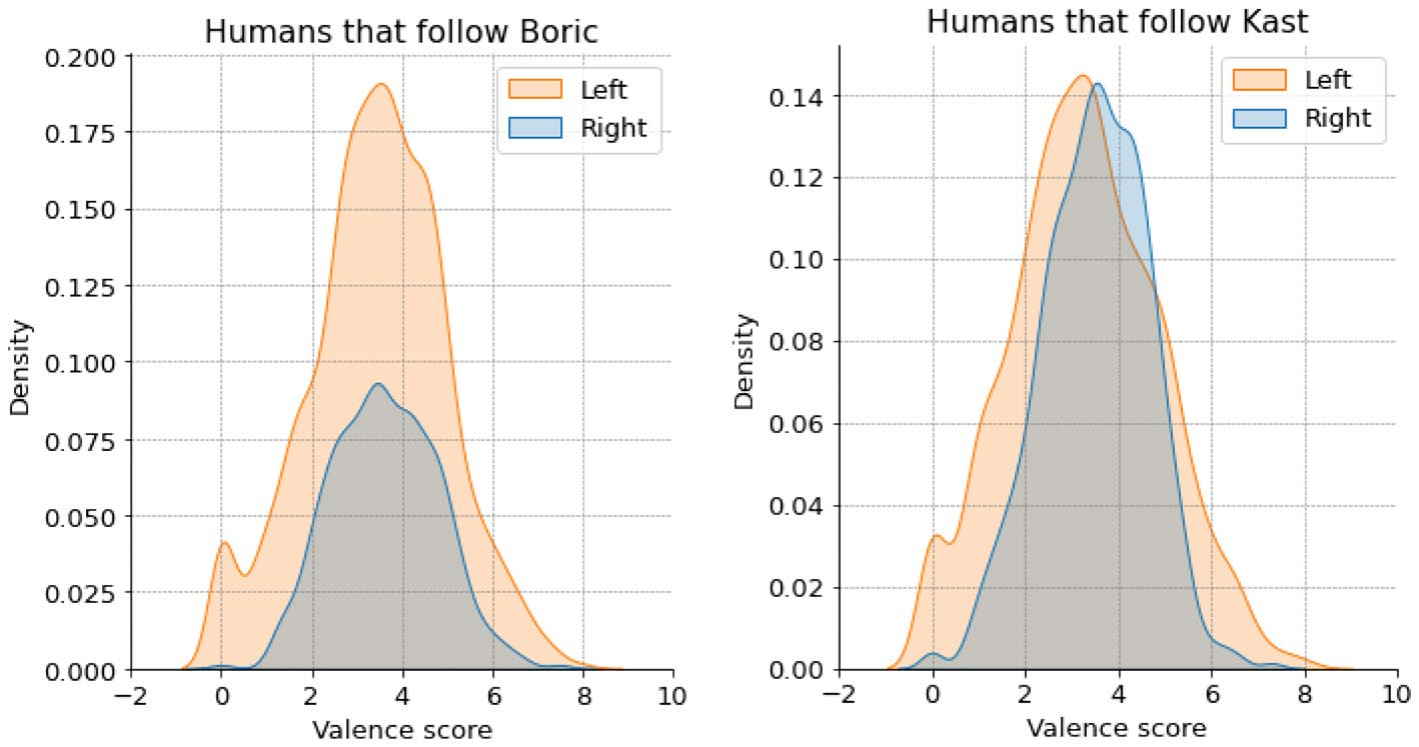
Valence scores (mean) per account (density plots). (Left) Humans that follow Gabriel Boric, and (Right) Humans that follow José Antonio Kast.

Figure 8 shows the distribution of the mean linguistic valence score of the bot accounts, disaggregated between right and left accounts. Figure 8 (left) indicates that bots on the right that follow Boric tend to use more negative language compared to those associated with the left. As depicted in Fig. 8 (right), it is observed that bots following Kast also exhibit negativity in their language. Among Kast’s followers, bots on the left demonstrate more positive language than those on the right, implying a tendency for left-aligned bots to use optimistic expressions. As the unpaired T-test reveals, statistically significant differences were found between the distributions of bots following Boric (mean left = 4.3, standard deviation = 1.8, mean right = 2.8, standard deviation = 1.9, p-value = 0.0001) and those following Kast (mean left = 2.2, standard deviation = 1.9, mean right = 0.9, standard deviation = 2.2, p-value = 0.0001). However, no significant differences were detected between the distributions of humans following Boric (see Fig. 9(left)) (mean left = 3.7, standard deviation = 2.2, mean right = 3.6, standard deviation = 1.9, p-value = 0.0596). In contrast, the differences are statistically significant among humans following Kast (see Fig. 9 (right)) (mean left = 3.4, standard deviation = 2.1, mean right = 4.0, standard deviation = 2.1, p-value = 0.0001).

Notably, while this analysis illustrates bot behavior patterns, both ideologically and emotionally, it is not conclusive about who creates and manages the bots. This remains an open question beyond the scope of the study.

## Conclusion

We have introduced Botcheck, a bot detector designed to detect bots in Twitter accounts whose primary language is Spanish. Botcheck outperforms other competitive methods in benchmark data. In addition, Botcheck pays close attention to the stylometry of the language used in the analyzed accounts, revealing clues to detect bots. In a case study using Botcheck, we detected the presence of bots in the accounts that follow the 2021 Chilean presidential candidates. Our analysis reveals differences in language usage between bots, which matches the type of campaign the candidates used.

### Limitations of this study

This study has limitations that are important to highlight so that future work can address them. First, the behavior of a bot detector strongly depends on the volume of annotated data on which the classifier can be trained, being the manual annotation process laborious and expensive. The work involved in the manual annotation of accounts limits the scale to which these datasets can reach, the main threat being the emergence of new types of bots, which, not being annotated in the dataset, could be difficult to detect by these methods. As demonstrated in our case study, Botcheck’s precision diminishes over time, since it relies on accounts annotated in Twibot-20, a process that extends up to the year 2020. When using accounts annotated in 2021, the method exhibits a decline in precision, even though it maintains a high level of recall. This outcome underscores the necessity of periodically updating the Botcheck model with newly annotated accounts, which will ensure the classifier remains updated. Another limitation is the impossibility of calculating network-based features from Twibot-20. This limitation prevented us from comparing ourselves with methods that operate globally, detecting botnets^25^. Instead, Botcheck operates at the local level, analyzing each account separately, without considering interactions between the accounts that reveal complex structures of coordination and synchronization of actions. Although this limitation is evident, it also causes Botcheck to work with features that are light to compute, favoring its use by accessing data retrieved from the Twitter API. Another limitation of the study is that the comparison of models based on their performance does not help explain why one model outperforms another. Our analysis indicates which are the most relevant features for Botcheck. However, a comprehensive comparison between models would allow for a comparative analysis, which was beyond the scope of this study. Finally, due to recent modifications in Twitter’s API usage policies, Botcheck no longer has access to the Twitter Academic API. Instead, it now utilizes the developer API of X, which imposes severe constraints on the number of allowable API calls. This restriction hampers the scale of analyses Botcheck can undertake.

## Data Availability

Researchers interested in using Twibot-20 should contact shangbin@cs.washington.edu to obtain permission to download the dataset for research purposes.
